# ‘Including us, talking to us and creating a safe environment’—Youth patient and public involvement and the Walking In ScHools (WISH) Study: Lessons learned

**DOI:** 10.1111/hex.13885

**Published:** 2023-10-06

**Authors:** Alison M. Gallagher, Sarah Maria O'Kane, Leanne C. Doherty, Maria Faulkner, Gary McDermott, Russell Jago, Ian M. Lahart, Marie H. Murphy, Angela Carlin

**Affiliations:** ^1^ Nutrition Innovation Centre for Food and Health (NICHE), Biomedical Sciences Research Institute Ulster University Coleraine UK; ^2^ Centre for Exercise Medicine, Physical Activity and Health, Sports and Exercise Sciences Research Institute Ulster University Belfast UK; ^3^ Institute of Nursing and Health Research Ulster University Londonderry UK; ^4^ Sports Lab North West Atlantic Technological University Donegal Letterkenny Ireland; ^5^ Population Health Sciences, Bristol Medical School University of Bristol Bristol UK; ^6^ Faculty of Education, Health and Wellbeing University of Wolverhampton Walsall UK; ^7^ Physical Activity for Health Research Centre (PHARC), Institute for Sport, Physical Education and Health Sciences University of Edinburgh Edinburgh UK

**Keywords:** adolescent girls, intervention development, patient and public involvement, physical activity, school

## Abstract

**Background:**

Young people have the right to be informed and consulted about decisions affecting their lives. Patient and public involvement (PPI) ensures that research is carried out ‘with’ or ‘by’ young people rather than ‘to’, ‘about’ or ‘for’ them. The aim of this paper is to outline how youth PPI can be embedded within a physical activity intervention, reflect on the impact of PPI and provide recommendations for future PPI in a similar context.

**Methods:**

A Youth Advisory Group (YAG) was set up within the Walking In ScHools (WISH) Study to involve adolescent girls in the delivery, implementation and dissemination of a physical activity intervention targeted at adolescents. Schools invited pupils aged 12–14 years and 15–18 years to YAG meetings (*n*3, from 2019 to 2023). Participative methods were used to inform recruitment strategies and data collection methods for the WISH Study.

**Results:**

Across the three YAG meetings, *n*51 pupils from *n*8 schools were involved. Pupils enjoyed the YAG meetings, felt that their feedback was valued and considered the meetings a good way to get young people involved in research. The YAG advised on specific issues and although measuring impact was not the primary aim of the YAG meetings, over the course of the study there were many examples of the impact of PPI. Recruitment targets for the WISH Study were exceeded, the attrition rate was low and pupils were engaged in data collection.

**Conclusion:**

Youth PPI is a developing field and there are few physical activity studies that report the PPI work undertaken. Within the WISH Study, three YAG meetings were held successfully, and the views of adolescent girls were central to the development of the study. Considering the specific issues that the YAG advised on (study recruitment, attrition and data collection), there was evidence of a positive impact of PPI.

**Patient or Public Contribution:**

Pupils from post‐primary schools interested/participating in the WISH Study were invited to attend YAG meetings. YAG meetings were set up to consult adolescent girls on the delivery, implementation and dissemination of the WISH intervention.

## 1 INTRODUCTION

Regular physical activity in adolescence is associated with beneficial health outcomes, including a reduced risk of obesity, improved physical fitness and cardiometabolic health, increased muscle and bone strength and a reduced risk of depression.^1^, ^2^, ^3^, ^4^ Despite this, it is estimated that worldwide, 81% of adolescents (aged 11–17 years) fail to meet the World Health Organisation recommendation of 60 min of moderate to vigorous physical activity per day.^5^, ^6^ The Global Action Plan on Physical Activity 2018–2030 recommends opportunities for physical activity should be integrated across multiple settings including schools.^5^ However, evidence has shown that previous school‐based efforts have had limited effects on physical activity and have not led to sustainable behaviour change.^7^, ^8^, ^9^, ^10^ Policymakers play a pivotal role in the design of physical activity interventions, but such interventions often have limited input from intervention recipients^11^ which can create a ‘policy gap’ in terms of understanding what young people need and want from interventions.^11^, ^12^

Young people have the right to be informed and consulted about decisions affecting their lives.^13^ Patient and public involvement (PPI) in research, refers to the inclusion of patients, carers, service users and stakeholders and may be defined as ‘research being carried out “with” or “by” members of the public rather than “to”, “about” or “for” them’ and is an integral part of the research process.^14^, ^15^ Young people can provide a unique perspective on the design, conduct and interpretation of research^16^ and PPI can enhance the quality, appropriateness, and relevance of health research.^17^ There are growing efforts to promote PPI, to recognise the importance of PPI as a necessary component of well‐designed research, and PPI has become a requirement and priority for many research funding bodies.^18^, ^19^ A recent scoping review reported the impact of youth PPI on research and researchers^20^ noting that PPI strengthened the design, conduct and rigour of research, and positively impacted researchers in terms of their skill development, knowledge and understanding of how to undertake PPI activity.^20^, ^21^, ^22^, ^23^, ^24^ There are numerous ways that young people can be involved in research ranging from consultation, design and development, analysis, and interpretation through to user‐led research.^25^ Youth Advisory Groups (YAGs) are often used for adolescents to work collaboratively with researchers at different stages of the research cycle.^25^ There are many variations of the YAG, and groups differ in terms of their membership, aims, objectives and methodology. Some groups are project‐specific while some contribute to multiple studies within an organisation.^26^ Being involved in PPI has many positive impacts on young people, namely on skill development, confidence, employment opportunities and creating positive change.^20^ Studies have reported that young people involved in PPI developed research and technical skills^21^, ^27^, ^28^, ^29^, ^30^ and an interest in pursuing a research career.^31^ Other studies report that young people developed their confidence,^28^, ^30^ advocacy skills^27^, ^28^, ^29^ and ability to work collaboratively.^32^

Despite the benefits of PPI for the research, researcher and young people involved,^20^ there is a lack of PPI reporting in physical activity research studies. It is essential that from the initial stages of the research process, the views of those expected to participate in the intervention are heard.^33^ Youth PPI has inherent challenges, for example, managing expectations, ensuring engagement is meaningful, and establishing effective working relationships with adult researchers.^32^ However, if we are to ensure that physical activity interventions for adolescents are relevant and appropriate, it is important that PPI is undertaken, documented, and evaluated.

The overall aim of this paper was to describe how adolescent girls have been involved for the delivery, implementation, and dissemination of a physical activity intervention (Walking In ScHools [WISH]) for adolescent girls. The specific objectives were to:

1. Present the PPI work undertaken and outline how a YAG was set up within a clustered randomised controlled trial (c‐RCT) (WISH Study).
2. Reflect on the impact of PPI activity in terms of the delivery and implementation of the wider WISH Study (recruitment, walk leader training, completeness of trial outcome data and COVID‐19 contingency plans).
3. Discuss the lessons learned based on the collective experiences gained from YAG meetings.
4. Provide an exemplar on how youth PPI can be undertaken and embedded within physical activity interventions.

## 2 METHODS

The WISH Study was a school‐based c‐RCT in girls aged 12–14 years from 18 schools across the border regions of Ireland and Northern Ireland. The study protocol was published in 2020 and the trial was registered prospectively with ISRCTN (ISRCTN12847782).^34^ The WISH Study was preceded by a mixed‐methods study that consulted adolescents to seek ideas on how to best promote physical activity among this population.^35^ The results of this study shaped the design of an effective feasibility study that increased light‐intensity physical activity.^36^ A mixed‐methods evaluation of the feasibility study was conducted with pupils and key stakeholders in participating schools to understand the experiences of those who participated and to assess the potential for schools to further promote physical activity outside of structured Physical Education. For the definitive WISH Trial, key elements of the study design were those already tested during the feasibility study.^36^ Therefore, the PPI work described in this manuscript relates to the YAG meetings and aspects of the delivery, implementation and dissemination of the WISH Study as opposed to the intervention design process. The GRIPP2 reporting checklist^37^ was completed (Supporting Information: File 1).

Ethical approval for the WISH Study (and trial outcomes reported in this manuscript) was granted by the Ulster University Research Ethics Committee (Reference: REC/19/0020); however, ethical approval was not required for the PPI element of the study as young people were involved as PPI contributors advising on the study conduct, refinement and dissemination rather than as research participants.^38^ The YAG contributions to discussion groups were not treated as research data but helped to make decisions that shaped the research study.^39^ Participation was voluntary and pupils were free to leave the YAG meetings at any time without giving a reason.

The initial plan was to conduct the WISH Study over a 2‐year period (Phase 1: nine schools involved from September 2019 to October 2020; Phase 2: nine schools involved from September 2020 to October 2021), considering the staff and resources available. However, due to the COVID‐19 pandemic and the closure of schools for face‐to‐face teaching, Phases 1 and 2 were suspended after the mean intervention duration of 16 and 8 weeks, respectively, as it was not possible to run the intervention or complete data collection. Subsequently, the fully powered trial was conducted with 18 schools in one phase from September 2021 to November 2022 (Phase 3).^40^ The trial outcome data (recruitment, data collection and walk leader training evaluation) presented in this paper are from Phase 3 of the WISH Study (September 2021 to November 2022) which was conducted as planned.

### 2.1 YAG

**Figure 1:**
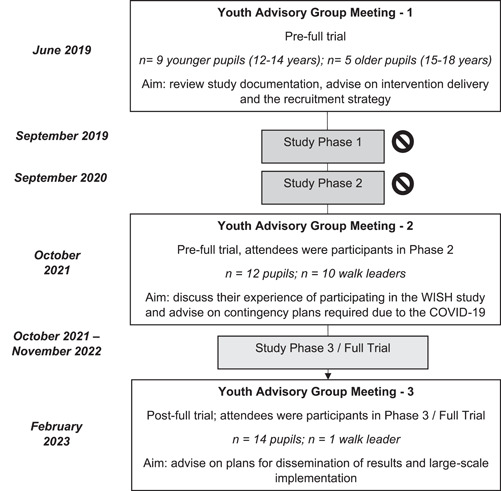
An overview of the key PPI activities undertaken. 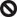 Indicates study suspension due to the COVID‐19 pandemic and associated school closures. PPI, patient and public involvement; WISH, Walking In ScHools.

The YAG was set up in the context of the WISH Study to consult adolescent girls on the delivery, implementation and dissemination of the WISH intervention and was based on the guidance of NIHR Centre for Engagement and Dissemination.^15^ As outlined in Figure 1, three YAG meetings occurred over a 4‐year period. Although Phases 1 and 2 of the WISH Study were suspended, the information generated and feedback received from YAG Meetings 1 and 2 were used to shape the delivery of the intervention (Phase 3) and will be discussed within this manuscript.

For each YAG meeting, to ensure that those participating in the YAG were of a similar age to trial participants and walk leaders, schools were asked to nominate two female pupils aged 12–14 years and two female pupils aged 15–18 years. Transport was arranged to bring attendees (and a member of school staff) to Ulster University. On arrival, attendees were provided with refreshments and an overview of the WISH Study in the form of a 10–15 min presentation delivered by the Trial Manager. Participative research methods were used, including discussion groups, games and creative activities to work collaboratively with the YAG members (Supporting Information: Table 1).

Before the discussion sessions at each meeting, the YAG participated in an ‘icebreaker’ activity (Supporting Information: Table 1). ‘Icebreakers’ can be effective at the beginning of a group session to help the leader become acquainted with participants and encourage participants to work together, get to know people from different backgrounds, and become involved in the session.^41^

At each YAG meeting, members were divided into smaller discussion groups (4–5 people), scheduled in 10–15 min slots and facilitated by the Research Team to work collaboratively and advise on specific issues (Supporting Information: Table 1). Parallel sessions were held with pupils aged 12–14 years and pupils aged 15–18 years. Facilitators (WISH Study research team members) were assigned to each discussion group to engage with the YAG, build rapport, be approachable and ensure the YAG felt valued and heard.^42^ For the interactive discussion sessions, the YAG were asked to discuss questions and there was flipchart paper on each table for contributors to record their ideas. At the end of each discussion session, the researcher with each group fed back their ideas allowing for clarification of points raised.^14^

At the end of each YAG meeting, attendees were asked to complete an anonymous evaluation survey (Supporting Information: File 2), the survey was estimated to take approximately 5 min to complete.

#### 2.1.1 YAG Meeting 1; June 2019; Pre‐Full Trial

Schools that had registered to participate in Phase 1 of the WISH Study (September 2019 to October 2020) were invited to attend the YAG meeting which was scheduled to last 4 h. The aim of this inaugural YAG meeting was to: (1) provide researchers with an understanding of what would encourage/discourage participation in the WISH Study; (2) refine the WISH Walk Leader Training Programme and (3) consult adolescent girls on study materials such as the fidelity checklist, accelerometer instructions and log. YAG members piloted the study questionnaires. All members of the YAG had the opportunity to design hooded sweatshirts (Supporting Information: Table 1) that would be provided as an incentive to participants in the WISH Study (pupils: for returning the accelerometer at the end of follow‐up data collection; walk leaders: for completing the walk leader training).

#### 2.1.2 YAG Meeting 2; October 2021; Pre‐Full Trial

Phase 2 of the WISH Study commenced in September 2020 but was suspended in February 2021 due to the COVID‐19 pandemic as schools were closed for face‐to‐face teaching and pupils moved to remote learning. We invited the schools (*n*2) that took part in Phase 2 of the WISH intervention to the second YAG meeting which was scheduled to last 3 h. Schools were asked to invite pupils (12–14 years) or walk leaders (15–18 years) who had participated in the study. The aim of this meeting was to gain feedback on the WISH intervention and to develop contingency plans should schools close for face‐to‐face teaching during Phase 3 to ensure that the intervention could continue if schools closed, and pupils returned to remote home learning. This meeting was held before Phase 3 (full trial) schools were randomised (control/intervention) and provided a unique opportunity to refine the intervention and seek ethical approval for any adjustments recommended by the YAG or required because of the pandemic and possible future school closures. At this meeting, the YAG were asked to complete a short 16‐item survey (Supporting Information: Files 3 and 4) about their experience of participating in the WISH Study before it was suspended due to the COVID‐19 pandemic. A second short six‐item survey (Supporting Information: Files 5 and 6) was administered to collect written feedback on the contingency plans. In total, the surveys were estimated to take no longer than 10 min to complete.

#### 2.1.3 YAG Meeting 3; February 2023; Post‐Full Trial

We invited a selection of schools that had been randomised to the intervention arm in Phase 3 of the WISH Study to the third YAG meeting which was scheduled to last approximately 3.5 h. The aim of this meeting was to discuss plans for the dissemination of study findings.

#### 2.1.4 The impact of PPI on the delivery and implementation of the WISH Study

The YAG advised on specific issues (namely recruitment, walk leader training, data collection and COVID‐19 contingency plans) and shaped the WISH physical activity intervention. It is difficult to determine the impact of PPI,^43^ and it cannot be reported with certainty that the outcomes reported in this paper are a direct result of PPI; however, these are the outcomes relating to the key issues that the YAG advised on. Key actions resulting from YAG meetings will be described alongside the following trial outcomes:

1. *Recruitment and attrition*: Recruitment of pupils and walk leaders was recorded at baseline. Retention of pupils was recorded at mid‐intervention; end of intervention and 12‐month follow‐up. Retention of walk leaders was recorded at the end of the intervention.
2. *Evaluation of walk leader training*: At the end of the training, walk leaders were asked to complete a short evaluation form that consisted of 14 questions.
3. WISH Study—Trial outcome measures:
  (a) *Accelerometer returns*: Records were kept of accelerometers issued and returned to the research team. Those not returned 4 weeks after 12‐month follow‐up data collection were classified as lost.
  (b) *Adherence to accelerometer wear‐time protocol*: The primary outcome measure of the WISH Study was total physical activity at the end of the intervention.^34^, ^40^ At four time points, pupils were asked to wear the accelerometer for 7 days. Pupils were included in the analysis if they had ≥2 valid weekdays of data.^34^, ^40^ The proportion of pupils meeting wear‐time criteria was recorded.
  (c) *Questionnaires*: For the main WISH Study analysis,^40^ pupils were asked to complete four questionnaires.^44^, ^45^, ^46^, ^47^, ^48^ The proportion of missing data was recorded.
4. *COVID‐19 contingency plans*: At the second YAG meeting, the pupils and walk leaders that were in the intervention group in Phase 2 of the WISH Study were asked to complete a short six‐item questionnaire (Supporting Information: Files 4 and 5) to help refine the study COVID‐19 contingency plans if there were further school closures due to the pandemic.

#### 2.1.5 Costs associated with the YAG meetings

The funding for the WISH Study began in April 2019 and concluded in May 2023. The project budget included funding of €4000 for the YAG meetings, representing <1% of the total grant budget. Costs associated with the YAG meetings (transport, catering and goody bags) were recorded and the average cost per meeting and cost per YAG member attending the meeting were calculated.

#### 2.1.6 Data analysis

Data were entered into SPSS for Windows (Version 28; SPSS Inc.) for analysis. Data entry and the coding of responses from the surveys were checked by a second researcher. Descriptive results will be reported and answers to open questions summarised.

### 2.2 Evaluation of the YAG meetings

Attendance at the YAG meetings was recorded. The research team/facilitators took notes throughout each discussion session. The data were analysed using the questions as a framework and key repetitive statements were counted and/or noted. Discussion notes were compiled and summarised. At the end of each meeting, attendees were asked to complete an anonymous paper‐based evaluation survey (Supporting Information: File 2). The survey consisted of 22 open and closed questions and were estimated to take no longer than 5 min to complete. Of the 22 questions, 16 were answered using a 7‐point scale (0–7), YAG members were asked to score from 0 to 7 how much they agreed with a set of statements and a higher score indicated a more positive review of the YAG meeting.

## 3 RESULTS

Across the three YAG meetings, a total of 51 pupils from eight schools were in attendance. Of the 14 schools invited to attend YAG meetings, 57% (*n*8) were able to participate. In total, *n*35 (69%) were pupils in Year 9/10 (12–14 years) and *n*16 (31%) were in Year 13/14 (15–18 years). The average attendance per meeting was 17 pupils. Less than a third (*n*14, 28%) of YAG members were involved in research before taking part in the WISH Study.

### 3.1 Evaluation of the YAG meetings

Across the three meetings, 51 evaluation surveys were received. A summary of the evaluation of YAG meetings is provided in Table 1. Participants were asked what they enjoyed most about the meeting and for *n*21 (41.2%) their response was categorised as enjoying the group work: ‘group talking, thinking of ideas’; designing the merchandise: ‘I enjoyed designing the half zips’; helping with the research: ‘I liked getting involved in giving feedback’; or learning more about the WISH Study: ‘Getting to know more about WISH and the people involved in it’. A quarter of YAG members (*n*13, 25.5%) stated that they enjoyed meeting new people: ‘Meeting new people, new ideas’, interacting with others: ‘Talking to the girls in the other school’ and meeting the research team: ‘The researchers were very nice and welcoming, they were easy to talk to’. Almost 10% of positive responses (*n*5, 9.8%) related to the catering: ‘Getting out together and working together and the scones’.

**Table 1:** Evaluation of the YAG meetings.

	*N*	Mean^a^	SD
I understand what the WISH study is about	51	6.37	1.10
I had the chance to give my feedback on the plans for the WISH study	51	6.51	0.99
I felt that my feedback was valued	51	6.45	1.05
I felt comfortable giving feedback to the researchers	51	6.43	1.08
It is important that young people have the chance to contribute to research studies	51	6.63	1.02
I enjoyed the presentation about the WISH study	51	6.12	1.07
I enjoyed the group work sessions	51	6.08	1.31
The catering was good	51	6.27	1.25
The meeting lasted about the right length of time	51	5.76	1.37
There was enough time for discussion	51	6.33	1.18
The researchers knew the subject well	51	6.63	1.06
The researchers helped everyone participate	51	6.73	0.96
This meeting was a good way of getting young people involved in research	51	6.43	1.10
I enjoyed today's meeting	51	6.41	0.98
Overall, I am glad that I attended the meeting	51	6.49	1.05
I would attend an event like this again	51	6.57	1.06

Abbreviations: SD, standard deviation; WISH Walking In ScHools; YAG, Youth Advisory Group.

^a^ Questions were answered using a 7‐point scale (0–7), with a higher score indicating a more positive review of the YAG meeting.

Pupils commented that the research team/facilitators had *‘*created a safe and positive atmosphere during the meeting*’* and the meeting was *‘*very well put together and I felt everyone in my group enjoyed the day*’*. When asked what they enjoyed least about the meetings, *n*27 YAG members provided a response, *n*6 (22.2%) stated that they did not like completing the questionnaires or that there was too much writing involved: *‘*Writing a lot’. Icebreaker activities were not enjoyed by *n*5 (18.5%) of those who provided a response: *‘*Talking in icebreaker*’* and *n*3 (11.1%) felt that the meetings involved prolonged sitting: *‘*Just sitting around talking*’*.

### 3.2 Key actions resulting from YAG meetings

Discussion notes from the YAG meetings are summarised in Tables 2 and 3.

**Table 2:** Key themes to emerge from YAG discussion groups with pupils (aged 12–14 years).

YAG Meeting 1; June 2019; Pre‐Full Trial	What would encourage you to take part in the WISH Study? Participating with friends Health benefits (sleep, mental health, fitness) Incentives/rewards
	What would not encourage you to take part in the WISH Study? Weather Missing lunchtime Low number of girls joining the walks
	What would you expect from your walk leaders? Organise and plan the walks Encourage participation (friendly, chat during the walks) Stamp reward cards fairly
	Incentive ideas Water bottles, keyrings, pens, highlighters, earphones, power bank, half zip jackets, speakers
	How can we encourage people your age to take part? Deliver a presentation to make people aware of the opportunity Incentives/rewards Emphasise the social aspect of the WISH Study
YAG Meeting 2; October 2021; Pre‐Full Trial	Did the walk leaders interact much with the younger girls during the walks? Walk leaders were described as encouraging, friendly and helpful The level of interaction varied across the schools
	Did the walk leaders always complete a checklist after the walks? The completion of checklists varied across schools
	Do you think the walks ran better at lunchtime or breaktime (or before school if this option was available? Lunchtime was the preferred option as there was more time to do a longer walk
	If this type of programme was to be introduced to your school, would you participate in the walks? The responses were varied but most YAG members said they would participate in the walks
	Did the incentives encourage you to take part in the walks? Yes, the incentives provided encouragement to join the walks
	Is there anything about the WISH programme that you think we could improve? Try to get more people to take part in the walks As the walking programme goes on, increase the value of incentives Include boys in the study
	Would you have liked to have stayed involved in WISH during lockdown when schools were closed for face‐to‐face teaching? It would have provided motivation to get active It would have helped maintain a healthy lifestyle
	Can you think of any benefits to being involved with the WISH Study during lockdown? Health benefits (emotional wellbeing, fitness, mental health) A distraction from school
	Can you think of any challenges to being involved with the WISH Study during lockdown? Time Weather Road safety
	What are your thoughts on the WISH team sending daily/twice daily text messages to remind girls to go for a walk if schools close for face‐to‐face teaching? Helpful reminder to go for a walk Some felt that reminders might be annoying
YAG Meeting 3; February 2023; Post‐Full Trial	Who should we communicate the findings of the WISH programme to? School community (teachers, Principals, pupils, parents) Government (health departments) and funding organisations Research scientists
	What messages do you think we should communicate to young people, now that the WISH Study is finished? Health benefits of walking (physical, mental health, fitness) Social aspects of the programme (meet new friends, socialise, reduce feelings of isolation) Incentives
	What do you think is the best method to communicate the findings of the WISH programme with schoolgirls? Social media (TikTok, Snapchat, Twitter, Facebook, Be Real, Whatsapp, Instagram) Influencers In schools (assemblies, posters, newsletters)
	Where would be the best place to communicate the findings of the WISH programme? Social media Schools Local media

**Table 3:** Key themes to emerge from YAG discussion groups with older pupils (aged 15–18 years).

YAG Meeting 1; June 2019; Pre‐Full Trial	What would encourage you to take part in the WISH Study? Recognition (badges, ‘walker of the month’, incentives) Improve fitness
	What would not encourage you to take part in the WISH Study? Weather Missing lunchtime/social time with friends Long walks (>10–15 min)
	What would you expect from your training? An understanding of how to work with young people Set the pace, routes and how to provide motivation Consensus that the training is called ‘WISH Walk Leader Award’
	Certificates, badges and heart rate monitors Want to be identifiable in school (hoodies, badges) Certificates after training and at the end of the intervention Would like to keep the heart rate monitors
	How can we encourage people your age to take part? Emphasise the opportunity for skill development Provide information on the training, what is required and incentives Refer to the ‘WISH Walk Leader Award’
YAG Meeting 2; October 2021; Pre‐Full Trial	Did you have any issues in running the programme in your school? Split lunchtimes Attendance Weather
	Were there any challenges to completing the checklists? Time required
	What would encourage you to complete the walk leader checklists? Set up a ‘Google Classroom’ Complete online while doing the walk Send reminders
	Do you think introducing incentives for the walk leaders would encourage completion of checklists? Yes, might encourage better compliance
	What do you think about having a ‘Walk Leader Champion’? Provide encouragement to other walk leaders and oversee the programme
	Would you have liked to have stayed involved in WISH during lockdown when schools were closed for face‐to‐face teaching? It would have provided motivation to get active It would have helped provide a sense of normality Enable contact with the younger girls
	Can you think of any benefits to being involved with the WISH Study during lockdown? Health benefits (emotional wellbeing, fitness, mental health) Reduce screen time Replace sports/clubs that were cancelled
	Can you think of any challenges to being involved with the WISH Study during lockdown? Road safety Communication challenges with the younger girls Time
	What are your thoughts on the WISH team sending daily/twice daily text messages to remind girls to go for a walk if schools close for face‐to‐face teaching? Helpful reminder to go for a walk Provide encouragement
YAG Meeting 3; February 2023; Post‐Full Trial	What messages do you think we should communicate to young people, now that the WISH Study is finished? Development of skills for walk leaders (communication, leadership, problem solving) Opportunity to meet new people Health benefits of walking
	Who should we communicate the findings of the WISH programme to? School community (teachers, Principals, pupils, parents) Government and county councils Researchers and healthcare professionals
	What do you think is the best method to communicate the findings of the WISH programme with schoolgirls? Social media In schools (Facebook page, posters) Influencers, sports people
	Where would be the best place to communicate the findings of the WISH programme? Social media Podcasts and local media (including health and wellbeing hubs) Schools (newsletter, Facebook page, school clubs)

Abbreviations: WISH, Walking In ScHools; YAG, Youth Advisory Group.

In terms of the key actions resulting from YAG meetings, the recruitment materials for the WISH Study were created using feedback from YAG members and focused on the health benefits of walking. To alleviate concerns about the weather, each intervention school was asked to create an indoor walking route (subject to available facilities). YAG members indicated that incentives were important and could help encourage participation and adherence to study protocols. As a result, incentives were provided when pupils returned accelerometers, these low‐cost incentives were chosen (water bottles, earphones, power banks) and designed (hooded sweatshirts) by YAG members. YAG members piloted the study questionnaires and recommended that these were completed electronically, Apple iPads® were used for data collection purposes. Based on the feedback from YAG members, each walk leader was provided with a badge for their school uniform and a WISH hooded sweatshirt with ‘Walk Leader’ printed on the back to wear during the walks to ensure that the younger pupils could identify the walk leaders within their school. Prizes were distributed for the walk leaders attending the most walks. When devising the walking timetable, walk leaders self‐selected the sessions they would lead and were able to partner with their friends. The WISH Walk Leader Award encompassed the guidance received from YAG members, information was provided on how to interact with and motivate younger pupils, how to set the pace of the walks and how to plan the route(s). Walk leaders were instructed to stamp reward cards fairly. Provision was made to ensure that the walk leaders could keep the heart rate monitors after the intervention was complete and certificates were issued following walk leader training. Recognising the challenges walk leaders faced in completing the fidelity checklists, an online version was made available for each intervention school in Phase 3 of the WISH Study. Younger pupils had the opportunity to nominate a ‘Walk Leader Champion’ within their school. Plans were devised to disseminate the results of the WISH Study with the key stakeholders identified by YAG members.

### 3.3 The impact of PPI on the delivery and implementation of the WISH study

#### 3.3.1 Recruitment and attrition

The YAG advised on what would encourage/discourage them from taking part in the WISH Study, they contributed to the recruitment strategy, helped create study recruitment materials, and informed the content delivered at recruitment sessions. The WISH Study aimed to recruit 384 pupils (average 24 pupils per school) and this target was exceeded by 53%. In total, 589 pupils were recruited (an average of 33 pupils per school), and 51 (8.7%) of pupils subsequently withdrew from the study. PPI shaped the development and refinement of the WISH Walk Leader Award and the number of walk leaders recruited (*n*149; *n*17 per school), exceeded the initial target of *n*135 or *n*15 per school by 10%. Over the intervention period, *n*4 (2.7%) of walk leaders withdrew from the study.

#### 3.3.2 Evaluation of WISH walk leader training

In total, 144 evaluation forms were completed. As outlined in Table 4, walk leaders felt positively about their training.

**Table 4:** Evaluation of the WISH Study walk leader training.

	Strongly agree		Agree		Disagree		Strongly disagree	
Item	*n*	%	*n*	%	*n*	%	*n*	%
The objectives of the training were met	122	84.7	22	15.3	0	0	0	0
The presentation was relevant	123	85.4	19	13.2	0	0	0	0
Participation and interaction were encouraged	119	82.6	24	16.7	1	0.7	0	0
The trainer was knowledgeable and delivered the training well	136	94.4	7	4.9	0	0	0	0
The content of the training was organised and easy to follow	127	88.2	17	11.8	0	0	0	0
The training session lasted the right length of time	118	81.9	25	17.4	1	0.7	0	0
The handouts were useful	122	84.7	22	15.3	0	0	0	0
I am glad I attended the training	127	88.2	17	11.8	0	0	0	0
I learnt something useful	118	81.9	24	16.7	2	1.4	0	0
I feel prepared to be a walk leader	123	85.4	20	13.9	0	0	0	0
I know what is required of me as a walk leader	126	87.5	17	11.8	1	0.7	0	0

*Note*: Total sample size *n* = 144.

Abbreviation: WISH, Walking In ScHools.

### 3.4 WISH study—Completeness of trial outcome measures

1. *Accelerometer returns*: At four time points, pupils were asked to wear the accelerometer for 7 days. In total, 2213 devices were issued over a 12‐month period. Only three (0.14%) devices were unreturned.
2. *Adherence to accelerometer wear‐time protocol*: The median overall wear time was 5–6 days at each time point. The number of pupils meeting the wear time criterion (≥2 weekdays) ranged from 91% (baseline) to 84% (end of intervention).
3. *Questionnaires*: At three time points, four questionnaires were administered for the main results paper.^40^ In total, 1656 responses were received. The proportion of missing data across the individual questionnaires ranged from 0% to 1.3%.

### 3.5 COVID‐19 contingency plans

In total, 22 responses were received for the COVID‐19 contingency plan questionnaire; 12 were from younger pupils and 10 were from walk leaders. The majority (*n*20; 91%) of YAG members would have liked to have stayed involved in the WISH Study when schools closed for face‐to‐face teaching during lockdown. Feedback from this questionnaire was used to refine COVID‐19 contingency plans for Phase 3.

### 3.6 Costs associated with YAG meetings

Costs associated with the YAG meetings (transport, catering, and resources) were recorded. The average cost per meeting was £951.06 and the average cost per YAG member attending the meeting was £54.63. Transport (average £501.33 per meeting) and catering (average £414.63 per meeting) costs when combined, accounted for 96% of the total spend.

### 3.7 Practical recommendations for research teams

Based on the challenges encountered and collective experiences gained through these YAG meetings, a series of practical recommendations for research teams are outlined in Table 5. These practical recommendations were reflections from the research team and based on experience of organising, conducting and evaluating youth PPI activities. Those involved as PPI facilitators had the opportunity to guide the practical recommendations.

**Table 5:** Practical recommendations for research teams based on the challenges encountered and collective experiences gained from YAG meetings.

Area	Recommendation
Planning	Where possible, provide transport and catering to widen access and participation. When inviting schools/pupils to attend the meetings, clearly outline to school staff what is required, for example, pupil numbers, time allocated and age/gender of pupils. Consider the recruitment process and devise a strategy to ensure that there is diversity among pupils attending meetings. Ensure a contingency plan is available should arrangements change on the day; a flexible approach is required when working with schools and young people. Carefully consider the catering options for young people and ensure the menu is suitable.
Communication	It is important to provide clear instructions to the young people involved and outline clearly what is expected of them. Remind YAG members that it is a safe space, there are no right or wrong answers, and everyone has something valuable to say. Consider devising some ‘ground rules’ ‘group values’ for YAG meetings, for example, be respectful of other people's opinions.
Meeting delivery	Facilitators should be experienced and able to involve all the young people in their group in each discussion session. Try to make meetings as interactive/fun as possible. Consider using games and icebreakers to help YAG members get to know each other. Provide written probes for facilitators to ensure the discussion flows. Keep discussion sessions short (10–15 min) to ensure members are engaged, attentive and focused. Include active breaks throughout the day to break up time spent sitting or sedentary.
Evaluation	Before undertaking PPI activity, devise and agree on an evaluation methodology. Consider capturing or evaluating the impact of the YAG meetings on the researchers involved. Use a log to formally record recommendations from PPI activities and if those recommendations were implemented. Consider if there is a need to inform YAG members of the impact of their contributions.

Abbreviations: PPI, patient and public involvement; YAG, Youth Advisory Group.

## 4 DISCUSSION

The current paper outlines how a YAG was set up within a c‐RCT (WISH Study) to undertake PPI to implement and disseminate a physical activity intervention for adolescent girls. There are few physical activity studies that report in any detail the PPI work undertaken with adolescent participants. This paper addresses a gap in knowledge and provides practical insights and recommendations for those conducting youth PPI.

Young people can be valuable partners in research as they can provide a unique perspective, on how research should be carried out.^16^, ^49^ The methods used for conducting PPI activities with adult populations may not be appropriate for young people and often there is a need to adapt existing methodologies.^50^ Youth PPI is a developing field and there have been limited reports of involving young people in the development of health interventions^20^ and in particular, physical activity interventions. Over the course of the WISH Study, we successfully held three YAG meetings with positive feedback from participating pupils, it was evident that pupils enjoyed attending the meetings. Pupils agreed that the YAG meetings were a good way of involving young people in research, were glad they attended the meeting and would attend a similar meeting in the future. However, there are challenges associated with youth PPI such as managing expectations, ensuring engagement is meaningful and establishing effective working relationships with adult researchers.^32^ The PPI process was reliant on school staff to invite pupils to meetings and to organise for pupils to leave school premises, which in our experience was challenging. Schools were supportive of the PPI activities undertaken and the wider WISH Study, however issues around timetabling, exam schedules and staffing meant that for some schools, it only became apparent on the morning of the YAG meeting that pupils were unable to attend. Good communication with school staff is important during the planning stages and it should be clearly outlined what is required in terms of pupil/teacher time and the of number of pupils to attend. Efforts should be made to check that arrangements are still suitable the day before the meeting, as often school plans can change at late notice despite careful planning.

The experience of facilitating PPI activity, provided some insight into the difficulties of maintaining engagement with adolescent girls and although this was a specific population group, the key findings reported are related to the PPI process and considered generalisable. It is important that all activities are engaging, fun and sustain interest, particularly during longer meetings. For future PPI activity, researchers should consider the duration of meetings, identify priority areas, and use participative methods to ensure participants remain engaged throughout the meeting. To ensure that all voices are heard and represented, it is important that we increase the diversity of people involved in PPI.^51^ In the present study, we were somewhat limited with the extent to which pupils from different schools could interact due to COVID‐19 restrictions and small numbers of pupils in attendance. Pupil recruitment to YAG meetings was managed at a school level and school staff selected the pupils to attend meetings. For future PPI activity, introducing a process where participants are randomly selected from school records, for example, and invited to attend may ensure greater diversity among YAG members.

Putting aside the challenges associated with youth PPI, there is potential for young people to contribute meaningfully to the implementation and dissemination of research studies.^20^ Over the course of the feasibility and definitive trial, the views of adolescent girls were instrumental in designing the WISH intervention.^35^ Within YAG meetings, adolescent girls were consulted on many aspects of the study, and discussions with YAG members were wide ranging. Although it is difficult to quantify the impact of PPI^43^ and there have been calls for more robust measures,^52^, ^53^ considering the issues that the YAG advised on specifically, a positive impact is evident. Recruitment targets for the WISH Study were exceeded and a low attrition/withdrawal rate was observed for both the younger pupils and walk leaders.^40^ Within the field of PPI, there have been calls for a more robust and critical evaluation^17^ and greater consistency in reporting of PPI impact.^54^ The impacts of PPI reported within this manuscript were based on retrospective observations of the specific issues that the YAG advised on and the associated outcomes. For future work, there is a need to plan systematically and develop validated tools to better understand the quality and full impact of PPI.^18^, ^43^

In our experience, PPI helped minimise the amount of missing trial outcome data. It is acknowledged that there are inherent challenges with using accelerometers to measure physical activity, particularly among adolescents such as compliance with wear time criteria,^55^ and non‐return of devices.^56^, ^57^ However, having addressed the feedback from the YAG on incentives for compliance with accelerometer wear‐time criteria, we observed that an exceptionally low number of accelerometers were unreturned (*n*3; 0.14%) and across four time points, >84% of pupils met wear‐time criteria.^55^, ^57^, ^58^ In the present study, the cost per YAG member attending the meeting was on average £54.63, but it is important to note that we did not capture researcher time, which would considerably increase the overall cost of this PPI work and would be an important consideration for future studies. For the YAG meetings outlined, members incurred no expenses; transport, meals and goody bags were provided. However, it is considered good practice to pay public contributors, including young people, for their time when involved in research and a remuneration and reward policy should be devised in advance of youth PPI activity.^59^, ^60^

There are many ways that young people can be involved in and contribute to PPI including consultation, involvement, collaboration and user‐led research.^20^ A novel approach by McQuinn et al.,^39^ was to use the Behaviour Change Wheel in combination with PPI to co‐design a school‐based physical activity intervention for adolescent females. Although the YAG was the most suitable approach for the WISH Study, given the time and resources available, it has been 9 years since the WISH feasibility study was conducted.^36^ In that time school environments have changed, primarily due to the COVID‐19 pandemic, in ways that would have been difficult to conceive when we designed the study and undertook the initial YAG meeting. Therefore, some elements of the WISH intervention may be more difficult to implement across the school context. There may be a need to rethink our approach to school‐based physical activity interventions and work with key stakeholders (pupils, teaching and support staff) to co‐produce and design appropriate interventions. Co‐production is ‘an approach in which researchers, practitioners and the public work together, sharing power and responsibility from the start to the end of the project, including the generation of knowledge’.^61^ To move the consultative PPI activity outlined in this manuscript towards co‐production, young people could be involved in setting priorities for research, data collection and analysis, developing tools and/or working collaboratively on study outputs.^30^, ^62^ In recent years, there has been growing interest in co‐production and these methods can enhance the effectiveness of an intervention by considering the perceived needs of the end user and the context within which an intervention will be delivered.^63^ While co‐production in physical activity research is relatively new and continually evolving,^64^ if we are to improve physical activity levels among adolescents, perhaps we need to reconsider a new approach to the design of school‐based interventions.

## 5 CONCLUSION

There is a need to increase physical activity levels among adolescents and engage with young people to design effective, relevant and acceptable interventions. The views of adolescent girls have been central to the development of the WISH Study and although youth PPI is not without its challenges, there are many benefits for researchers, the study and the young people involved. The practical recommendations outlined in this paper will help research teams harness the potential that exists for young people to contribute meaningfully to the design, conduct and dissemination of research.

## AUTHOR CONTRIBUTIONS

Alison M. Gallagher, Sarah Maria O'Kane, Leanne C. Doherty, Maria Faulkner, Russell Jago, Ian M. Lahart, Marie Murphy and Angela Carlin conceptualised the study and defined the methodology. Funding was acquired by Marie H. Murphy, Alison M. Gallagher, Angela Carlin, Maria Faulkner, Russell Jago and Ian M. Lahart. Sarah Maria O'Kane and Ian M. Lahart conducted the data analysis. Sarah Maria O'Kane, Leanne C. Doherty and Gary McDermott were responsible for the project administration. Sarah Maria O'Kane prepared the original manuscript which was reviewed and edited by all authors.

## CONFLICT OF INTEREST STATEMENT

The authors declare no conflict of interest.

## ETHICS STATEMENT

Ethical approval for the Walking In ScHools Study was granted by the Ulster University Research Ethics Committee (Reference: REC/19/0020) on June 20, 2019, although ethical approval was not required for the patient and public involvement (PPI) element of the study as young people were involved as PPI contributors advising on the study design, refinement and dissemination rather than being research participants.^38^ Participation was voluntary and pupils were free to leave the Youth Advisory Group meetings at any time without giving a reason.

## Supporting information

Supporting information.

Supporting information.

Supporting information.

Supporting information.

Supporting information.

Supporting information.

Supporting information.

Supporting information.

## Data Availability

The datasets generated and/or analysed for the current study are not publicly available at present but are available from the corresponding author at reasonable request.
