# Supplementary material for: ‘Including us, talking to us and creating a safe environment’—Youth patient and public involvement and the Walking In ScHools (WISH) Study: Lessons learned
Source: Health Expect. 2023 Oct 6;27(1):e13885. doi: 10.1111/hex.13885 (PMC10726144; doi:10.1111/hex.13885)
Supplement: Supplementary file 1 — Supporting information. [file HEX-27-e13885-s001.docx]

| **Section and topic** | **Item** | **Reported on page number** |
| --- | --- | --- |
| 1: Aim | Report the aim of PPI in the study | 5 |
| 2: Methods | Provide a clear description of the methods used for PPI in the study | 5-12 |
| 3: Study results | Outcomes—Report the results of PPI in the study, including both positive and negative outcomes | 13-17; 32-37 |
| 4: Discussion and conclusions | Outcomes—Comment on the extent to which PPI influenced the study overall. Describe positive and negative effects | 18-22 |
| 5: Reflections/critical perspective | Comment critically on the study, reflecting on the things that went well and those that did not, so others can learn from this experience | 18-22; 38 |

**Supplementary File 1:** Guidance for Reporting Involvement of Patients and the Public (GRIPP2) Reporting Checklist (short form)^1^

1. Staniszewska S, Brett J, Simera I, et al. GRIPP2 reporting checklists: Tools to improve reporting of patient and public involvement in research. *Res Involv Engagem*. 2017;3(1):1-11. doi:10.1186/S40900-017-0062-2/TABLES/4
