# Supplementary material for: ‘Including us, talking to us and creating a safe environment’—Youth patient and public involvement and the Walking In ScHools (WISH) Study: Lessons learned
Source: Health Expect. 2023 Oct 6;27(1):e13885. doi: 10.1111/hex.13885 (PMC10726144; doi:10.1111/hex.13885)
Supplement: Supplementary file 2 — Supporting information. [file HEX-27-e13885-s007.docx]

**Supplementary File 3:** Experience as a WISH Study - participant survey for pupils (12-14 years)


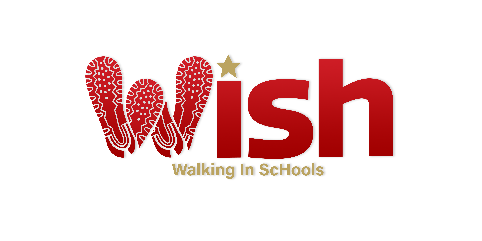


***Youth Advisory Group, 15^th^ October 2021***

***Session 1: Participants (12-14yrs) Questionnaire***

**Why did you sign up to the WISH Study?**

**On average, how many times a week did you go on the walk?**

- Less than once a week
- 2-4 times per week
- 5-7 times per week
- 8-10 times per week

**Do you think the walking programme ran well in your school?**

- Yes
- No

*Please state the reason for your answer:*

**Were the walks enjoyable?**

- Yes
- No

*Please state the reason for your answer:*

**Would you have liked the teacher to be more or less involved?**

- More involved
- Less involved
- They were involved the correct amount

*Please state the reason for your answer:*

**What did you like about the WISH Study?**

**Was there anything about the WISH Study that you didn’t like?**

- Yes
- No

*Please state the reason for your answer:*

**Do you think themed walks would encourage the girls to go out on the walks? For example, Santa Hat walks at Christmas, Easter Hunt etc.**

- Yes
- No

*Please state the reason for your answer:*

**Was there always a walk leader present to lead the walks?**

- Yes
- No

**Did you find the walk leaders easy to talk to?**

- Yes
- No

**Did you find the walk leaders supportive or encouraging?**

- Yes
- No

**Do you think the walk leaders ran the walking programme well?**

- Yes
- No

*Please state the reason for your answer:*

**How did you find walking in a group?**

**Were there any issues with the walk leaders?**

- Yes
- No

*Please state the reason for your answer:*

**Were there any issues in getting your reward for going on the walks?**

- Yes
- No

*Please state the reason for your answer:*

**Would you take part in this type of programme/research study again?**

- Yes
- No

*Please state the reason for your answer:*

**Thank you for taking the time to complete the questionnaire**
