# Supplementary material for: ‘Including us, talking to us and creating a safe environment’—Youth patient and public involvement and the Walking In ScHools (WISH) Study: Lessons learned
Source: Health Expect. 2023 Oct 6;27(1):e13885. doi: 10.1111/hex.13885 (PMC10726144; doi:10.1111/hex.13885)
Supplement: Supplementary file 3 — Supporting information. [file HEX-27-e13885-s008.docx]

**Supplementary File 2:** YAG Evaluation Survey


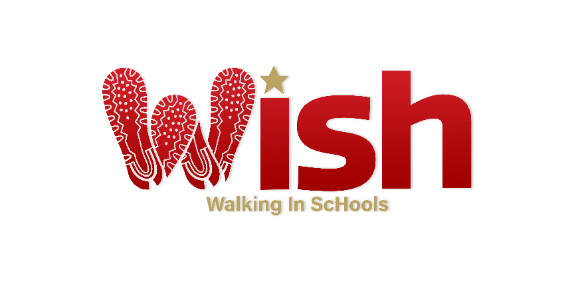


**Thank you very much for joining us today for the WISH Youth Advisory Group Meeting. We are really grateful for your participation, ideas and feedback on the WISH study plans.**

Please take a few minutes to complete this survey.

**Are you in:**

| Year 9/10 (NI) or 1^st^ /2nd YR (ROI) |  |
| --- | --- |
| Year 13/14 (NI) or TY/6^th^ form (ROI) |  |

**Have you been involved in a research study before taking part ?**

| Yes |  |
| --- | --- |
| No |  |
| Don’t know |  |

**What did you enjoy most about today’s meeting?**

**What did you enjoy least about today’s meeting?**

**In future, is there anything you think the WISH team should change?**

**Please circle one number for each question using the following scale: 1 (strongly disagree) to 7 (strongly agree).**

|  | **Strongly disagree** | | |  | | | **Strongly agree** | | |
| --- | --- | --- | --- | --- | --- | --- | --- | --- | --- |
| I understand what the WISH study is about | 1 | 2 | 3 | | 4 | 5 | | 6 | 7 |
| I had the chance to give my feedback on the plans for the WISH study | 1 | 2 | 3 | | 4 | 5 | | 6 | 7 |
| I felt that my feedback was valued | 1 | 2 | 3 | | 4 | 5 | | 6 | 7 |
| I felt comfortable giving feedback to the researchers | 1 | 2 | 3 | | 4 | 5 | | 6 | 7 |
| It is important that young people have the chance to contribute to research studies | 1 | 2 | 3 | | 4 | 5 | | 6 | 7 |
| I enjoyed the presentation about the WISH study | 1 | 2 | 3 | | 4 | 5 | | 6 | 7 |
| I enjoyed the group work sessions | 1 | 2 | 3 | | 4 | 5 | | 6 | 7 |
| The catering was good | 1 | 2 | 3 | | 4 | 5 | | 6 | 7 |
| The meeting lasted about the right length of time | 1 | 2 | 3 | | 4 | 5 | | 6 | 7 |
| There was enough time for discussion | 1 | 2 | 3 | | 4 | 5 | | 6 | 7 |
| The researchers knew the subject well | 1 | 2 | 3 | | 4 | 5 | | 6 | 7 |
| The researchers helped everyone participate | 1 | 2 | 3 | | 4 | 5 | | 6 | 7 |
| This meeting was a good way of getting young people involved in research | 1 | 2 | 3 | | 4 | 5 | | 6 | 7 |
| I enjoyed today’s meeting | 1 | 2 | 3 | | 4 | 5 | | 6 | 7 |
| Overall, I am glad that I attended the meeting | 1 | 2 | 3 | | 4 | 5 | | 6 | 7 |
| I would attend an event like this again | 1 | 2 | 3 | | 4 | 5 | | 6 | 7 |

**Is there anything else you would like to say about today’s meeting?**

***Thank you for taking the time to complete this survey!***
