# Supplementary material for: ‘Including us, talking to us and creating a safe environment’—Youth patient and public involvement and the Walking In ScHools (WISH) Study: Lessons learned
Source: Health Expect. 2023 Oct 6;27(1):e13885. doi: 10.1111/hex.13885 (PMC10726144; doi:10.1111/hex.13885)
Supplement: Supplementary file 4 — Supporting information. [file HEX-27-e13885-s003.docx]

**Supplementary File 4:** Experience as a WISH Study - participant survey for walk leaders (15-18 years)


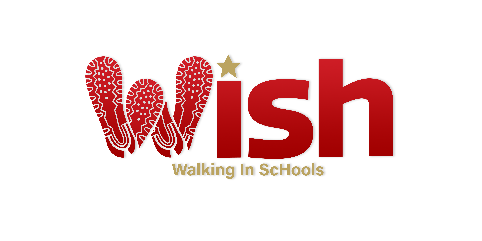

***Youth Advisory Group, 15^th^ October 2021***

***Session 1: Walk leader (15-18 yrs) Questionnaire***

**Why did you sign up to be a walk leader?**

**What did you think was required of you as a walk leader?**

**On average, how many times a week did you lead a walk?**

- Less than once a week
- Once a week
- Twice a week
- Three times per week
- Four or more times per week

**Were there any issues that stopped you attending the walks?**

*Please state the reason for your answer:*

**What do you feel you got out of being a walk leader on the WISH study?**

**Do you think the walking programme ran well in your school?**

- Yes
- No

*Please state the reason for your answer:*

**Would you have liked the teacher to be more or less involved with running the programme?**

- More involved
- Less involved
- They were involved the correct amount of time

*Please state the reason for your answer:*

**Did you have any issues in running the walking programme in your school?**

- Yes
- No

*Please state the reason for your answer:*

**Do you think the younger girls were engaged in the walking programme?**

- Yes
- No

*Please state the reason for your answer:*

**Can you think of ways to engage the younger girls in the walking programme?**

**Do you think themed walks would encourage the girls to go out on the walks? For example, Santa Hat walks at Christmas, Easter Hunt etc.**

- Yes
- No

*Please state the reason for your answer:*

**Do you think anything should be changed to improve the WISH walking programme?**

- Yes
- No

*Please state the reason for your answer:*

**Do you think the WISH walking programme is something all schools should implement?**

- Yes
- No

*Please state the reason for your answer:*

**Did you engage with the walk leader facebook page?**

- Yes
- No

*Please state the reason for your answer:*

**Can you suggest a better alternative to Facebook that people your age would use?**

**What information would you like the WISH team to include on facebook (or alternative platform)?**

**Thank you for taking the time to complete the questionnaire**
