# Supplementary material for: ‘Including us, talking to us and creating a safe environment’—Youth patient and public involvement and the Walking In ScHools (WISH) Study: Lessons learned
Source: Health Expect. 2023 Oct 6;27(1):e13885. doi: 10.1111/hex.13885 (PMC10726144; doi:10.1111/hex.13885)
Supplement: Supplementary file 5 — Supporting information. [file HEX-27-e13885-s004.docx]

**Supplementary File 5:** Survey on WISH Study COVID-19 Contingency Plan for pupils (12-14 years)


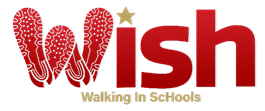


***Youth Advisory Group, 15^th^ October 2021***

**Session 2: Participants *(12-14yrs)*Questionnaire**

**WISH contingency plan**

**Would you liked to have stayed involved with the WISH programme when schools closed for** **face-to-face teaching during lockdown?**

- Yes
- No

*Please state the reason for your answer:*

**Do you think it would be a good idea for the WISH team to send daily/twice daily messages to WISH participants, reminding them to go for a walk, if schools close for face-to-face teaching?**

- Yes
- No

*Please state the reason for your answer:*

**Do you think it would be a good idea to have a weekly video call, so that the walk leaders could encourage the younger girls to go for a walk, if schools close for face-to-face teaching?**

- Yes
- No

*Please state the reason for your answer:*

**How often do you think the younger girls and walk leaders should have a video call if schools close for face-to-face teaching?**

- Once a fortnight
- Once a week
- 2-3 times a week
- Other please state ____________________________________________________________

**What would be the best platform to use for a video call?**

**Do you think it would be a good idea for a member of the WISH team to join the video calls?**

- Yes
- No

*Please state the reason for your answer:*

**Thank you for taking the time to compete the questionnaire**
