# Supplementary material for: ‘Including us, talking to us and creating a safe environment’—Youth patient and public involvement and the Walking In ScHools (WISH) Study: Lessons learned
Source: Health Expect. 2023 Oct 6;27(1):e13885. doi: 10.1111/hex.13885 (PMC10726144; doi:10.1111/hex.13885)
Supplement: Supplementary file 7 — Supporting information. [file HEX-27-e13885-s002.docx]

**Table S1:** Participatory research methods used within PPI activities

| ***Activity*** | ***Details*** |
| --- | --- |
| *Ice breaker games* | *Name bingo (YAG Meeting One)*  Members were given a sheet with fifteen statements and were asked to move around the room and find a person that matches a fact. They were asked to write the person’s name down and the first person to have written a name against each of the statements, shouted “bingo” and were deemed the winner. Examples of statements included: “Someone who has lived in another county”; “Someone who plays a team sport”; “Someone who can play an instrument”  *Walking bingo (YAG Meeting Two)*  Members were given a sheet with twelve statements and were asked to move around the room and find a person that matches a fact. They were asked to write the person’s name down and the first person to have written a name against each of the statements, shouted “bingo” and were deemed the winner. Examples of statements included: “Walks their dog regularly”; “Prefers to walk on the beach”; “Enjoys listening to music while walking”  *Toilet paper speaking activity (YAG Meeting Three)*  A roll of toilet paper was placed in the centre of each table, YAG members were asked to take the roll of toilet paper, pull off several squares before handing it to the next person and asking them to do the same. Once all members had some toilet paper, members were asked to count the number of squares they had and then tell their group that number of facts/things about themselves. For example, if someone took four squares of toilet paper, they would be asked to share four things about themselves. |
| *Creative activities* | *Design a WISH hoodie (YAG Meeting One)*  Members were given a paper template of a sweatshirt. This was blank and members were provided with small cut outs of the two logos to be placed on the hoodies (WISH and Ulster University). In this creative activity, members were given art supplies and asked to design a sweatshirt that could be provided to study participants as an incentive. |
| *Discussion groups* | Interactive discussion sessions (similar format across three meetings)  The YAG were asked to discuss topics/questions in smaller discussion groups that lasted 10-15mins in duration. On each table were markers, sticky notes, and flipchart paper. As topics/ideas were being discussed, members were asked to write these on sticky notes and place them on the flipchart paper. At the end of the discussion, the facilitators within each group fed back their answers/ideas. The flipchart paper containing the ideas on sticky notes were placed on the walls around the room to generate further discussion and to record the points made. |
