# Supplementary material for: ‘Including us, talking to us and creating a safe environment’—Youth patient and public involvement and the Walking In ScHools (WISH) Study: Lessons learned
Source: Health Expect. 2023 Oct 6;27(1):e13885. doi: 10.1111/hex.13885 (PMC10726144; doi:10.1111/hex.13885)
Supplement: Supplementary file 8 — Supporting information. [file HEX-27-e13885-s005.docx]

Supplementary Table 2: Topics for group discussion

| YAG Meeting One; June 2019; Pre-full trial | Pupils (12-14 years) | 1) What would encourage you to take part in the WISH Study?  2) What would not encourage you to participate in the WISH Study?  3) What would you expect from your walk leaders?  4) What incentives could be provided for returning the accelerometer?  5) Review of questionnaires to be used within the main trial evaluation  6) Review of accelerometer guidelines and log  7) How can we encourage people your age to take part? |
| --- | --- | --- |
|  | Pupils (15-18 years) | 1) What would encourage you to take part in the WISH Study?  2) What would discourage you from participating in the WISH Study?  3) What would you expect from your training and what would you prefer for the training to be called?  4) What incentives could be provided for volunteering as a walk leader?  5) Walk Leader’s Facebook Group – is Facebook the best platform for a closed social media group and what type of content should be posted?  6) Review of walk leader fidelity checklist  7) How can we encourage people your age to take part? |
| YAG Meeting Two; October 2021; Pre-full trial | Pupils (12-14 years) | 1) Did the walk leaders interact much with the younger girls during the walks? 2) Did the walk leaders get to know the younger girls?  3) Did the walk leaders always complete a checklist after the walks?  4) Do you think the walks ran better at lunchtime or breaktime (or before school if this option was available)?  5) If this type of programme was to be introduced to your school, would you participate in the walks?  6) Did the incentives encourage you to take part in the walks?  7) Is there anything about the WISH programme that you think we could improve?  8) Would you have liked to have stayed involved in WISH during lockdown when schools were closed for face-to-face teaching?  9) Can you think of any benefits to being involved with the WISH Study during lockdown?  10) Can you think of any challenges to being involved with the WISH Study during lockdown?  11) What are your thoughts on the WISH team sending daily/twice daily text messages to remind girls to go for a walk if schools close for face-to-face teaching? |
|  | Pupils (15-18 years) | 1) Did you have any issues running the walking programme in your school?  2) Were there any challenges to completing the checklists? 3) What would encourage you to complete the walk leaders checklists?  4) Do you think introducing incentives for the walk leaders would encourage completion of the checklists?  5) What do you think about having a Walk Leader Champion?  6) Would you have liked to have stayed involved in WISH during lockdown when schools were closed for face-to-face teaching?  7) Can you think of any benefits to being involved with the WISH Study during lockdown?  8) Can you think of any challenges to being involved with the WISH Study during lockdown?  9) What are your thoughts on the WISH team sending daily/twice daily text messages to remind girls to go for a walk if schools close for face-to-face teaching? |
| YAG Meeting Three; February 2023; Post-full trial | Pupils (12-14 years) | 1) Who should we communicate the findings of the WISH programme to?  2) What messages do you think we should communicate to young people, now that the WISH Study is finished? 3) What do you think is the best method to communicate the findings of the WISH programme with schoolgirls? 4) Where would be the best place to communicate the findings of the WISH programme? |
|  | Pupils (15-18 years) | 1) What messages do you think we should communicate to young people, now that the WISH programme is finished?  2) What do you think is the best method to communicate the findings of the WISH programme with schoolgirls?  3) Where would be the best place to communicate the findings of the WISH programme?  4) Who should we communicate the findings of the WISH study to? |
